# Overcoming Access Barriers for Facility-based Delivery in Low-income Settings: Insights from Bangladesh and Uganda

**Published:** 2006-12

**Authors:** Justin O. Parkhurst, Syed Azizur Rahman, Freddie Ssengooba

**Affiliations:** ^1^ Health Policy Unit, London School of Hygiene & Tropical Medicine, Keppel Street, London WC1E 7HT, United Kingdom; ^2^ Institute of Public Health, Makerere University, New Mulago Hospital Complex, PO Box 7072, Kampala, Uganda

**Keywords:** Maternal health, Delivery, Health services, Healthcare, Health facilities, Comparative studies, Uganda, Bangladesh

## Abstract

Women in both Bangladesh and Uganda face a number of barriers to delivery in professional health facilities, including costs, transportation problems, and sociocultural norms to deliver at home. Some women in both the countries manage to overcome these barriers. This paper reports on a comparative qualitative study investigating how some women and their families were able to use professional delivery services. The study provides insights into the decision-making processes and overcoming access barriers. Husbands were found to be particularly important in Uganda, while, in Bangladesh, a number of individuals could influence care-seeking, including unqualified local healers or traditional birth attendants. In both the settings, cost and transport barriers were often overcome through social networks. Social prohibitions on birth in the health facility did not feature strongly in women's accounts, with several Ugandan women explaining that friends or peers also used facilities, while, in Bangladesh, perceived complications apparently justified the use of professional medical care. Investigating the ways in which some women can overcome common barriers can help inform policy and planning to increase the use of health facilities for child delivery.

## INTRODUCTION

Increasing the proportion of women who deliver in a health facility can be an important means in reducing maternal mortality in low-income settings. It is globally recognized that one of the main challenges to achieving the Millennium Development Goal (MDG) of a global reduction of maternal death by 75% by 2015 (1) is the low proportion of women who deliver with a skilled birth attendant (2). Deliveries in health facilities can ensure that women are attended by skilled personnel and also link women to the referral system in the case of any complications. In many low-income settings with a high burden of maternal deaths, few women use facilities for birth, often choosing a higher-risk birth at home, often without professional medical assistance. Past studies have identified several reasons why women or families may choose home-delivery as a preferred option and a number of access barriers to using facilities as well. In all countries, there are some women who choose to deliver in health facilities and who are seemingly able to overcome the known access barriers.

This study presents findings of an in-depth investigation into users of professional delivery services in two countries—Uganda and Bangladesh—to learn more about how to encourage the use of facilities and enable women and their families to overcome barriers they may face in doing so. The paper presents comparative results from women's accounts of their own pregnancy and delivery-related experiences.

### Background

Uganda and Bangladesh are both low-income countries, facing similar reported levels of maternal mortality, which also represent very different national contexts. Uganda is an East African nation of mixed religious and ethnic backgrounds. It obtained independence from Britain in 1962 but was soon engulfed by civil wars that lasted until 1986, when the current government took power and brought stability to most of the country. The civil wars destroyed much of the health infrastructure (3), and the country has been slowly rebuilding since its end (4, 5). Bangladesh gained independence in 1971 after a liberation war with Pakistan. The country is larger in population than Uganda, but is culturally more homogeneous, with a large majority Bangla-speaking Muslim population. The governments of both the countries are working towards MDG-related outcomes, including reducing maternal death, with some ongoing strategies in place, such as a current WHO-sponsored initiative to train community-based skilled birth attendants in Bangladesh (6, 7). Table 1 outlines some socioeconomic and health service features of the two countries—as shown, both the countries have a low use of skilled attendants for child delivery and high maternal mortality.

**Table 1.** Uganda and Bangladesh comparison of key figures

Variable	Uganda	Bangladesh
Population (million), 2002*	25.0	143.8
GNP per capita, 2002 (US$)*	236	351
Human development ranking, 2002*	146	138
Physicians per 100,000 people†	4.7	23.0
Nurses and midwives per 100,000 people† (8)	33.4	24.0
Proportion (%) of births attended by skilled health personnel*	39	12.1
Maternal mortality ratio (deaths/100,000 livebirths) (9, 10)	505	320

*UNDP country statistics (www.undp.org, accessed on 5 August 2005);

†WHO global atlas of the health workforce (www.who.int/globalatlas/default.asp, accessed on 30 September 2005);

GNP=Gross national product;

UNDP=United Nations Development Programme

### Health-seeking behaviour

There are several reasons behind the low use of professional services in these countries, and a number of country-specific studies have identified potential barriers to access which can lead to home-delivery, either unattended or with traditional birth attendants (TBAs) in the home. The most common barriers mentioned include: distance from a health facility, transportation problems, costs of services, including informal charges or expenses, opportunity costs from time lost, perceived low-quality care in facilities, or cultural barriers to professional health-seeking, including stigma, fear, inability for women to travel alone, or to be seen by male doctors (11–15). Many of these barriers are not country-specific, as the barriers of distance, costs, demands on women's time, and lack of decision-making power in the household have all been identified internationally as key reasons for which women do not seek professional maternal health services in a timely manner (16, 17).

In addition, studies in both the countries have identified social norms for childbirth to be seen as a normal event, or even a test for women to endure on their own. In Bangladesh, results of an anthropological study showed that blame was placed on women who needed to ask for assistance or have delivery in a facility, as they were believed to have done something to cause the difficulties which lead to the problems requiring assistance (13). Similarly in Uganda, a focus-group study presented the opinion that abnormal pregnancies should only be handled in facilities (8), while another study highlighted a common view of pregnancy as dangerous, but also found “the woman who delivers herself was said to be highly respected” (18).

Despite all of the known barriers and pressure to deliver at home, there are still some women in both the countries who do deliver their children in health facilities, even in poor rural communities. This paper attempts to understand the experiences of some of these women through in-depth interviews to know how decisions were made and barriers overcome. Many past qualitative studies looking at norms of child delivery have focused on describing the norms of non-use of services, rather than factors leading to use. The existing studies focussing on users of services have tended to be quantitative surveys, identifying statistical correlates of use, including sociodemographic elements, such as age, income, or education. These quantitative studies have also found important factors, such as use of antenatal care (19) or experience of complications/abnormal pregnancy (11, 20) to be key factors determining the use of professional delivery services. Such studies cannot illustrate how decisions are made, or the key enabling factors behind these decisions.

## MATERIALS AND METHODS

This qualitative study focusing on service-users was conducted to learn more about the decision-making process for women, and how women and families act to overcome access barriers for delivery-care. The study was conducted in one district in both Bangladesh and Uganda. In each country, 30 women from across the district, who had recently delivered in a health facility, were interviewed with the profile of women included (Table 2).

**Table 2.** Background characteristics of interviewed women

Characteristics	Uganda (n=30)	Bangladesh (n=30)
Age (years) range	17–37	16–35
Number of pregnancies	
1	11	11
2	17	6
3	1	6
4+	1	7
Education	
None	4	10
Some Primary	9	5
Primary	8	6
Some Secondary	4	8
Secondary	4	1
Higher education	1	0
Religion	
Muslim	2	23
Hindu	0	6
Protestant	9	0
Catholic	13	0
Other (or unspecified) Christian	3	1
Other	3	0
Employment	
House work/consumption agriculture	14	29
Agriculture to sell	10	0
Trader/shop work	4	0
Wage employed	2	1

Interviews in Uganda were conducted in Hoima district, located on the western border with the Democratic Republic of Congo. The district has a predominantly rural settlement typical for the majority of the population of Uganda and has a population of approximately 401,000, who depend mostly on agriculture, with tea and tobacco as the main crops for income. The healthcare system consists of a medium-sized hospital of 280 beds and one health centre with a capacity for emergency obstetric care. In addition, 28 smaller health centres provide services for uncomplicated deliveries, antenatal care, family planning, and essential clinical-care services. The district has a hilly geographic terrain and a precarious road infrastructure, especially during wet weather. Women were purposively selected to achieve a spread across areas of the district.

The work in Bangladesh was conducted in Jhenaidah district located in the southwest of the country, over 200 km from the capital city of Dhaka. The district has a population of over 1.5 million and is divided into five upazilas (sub-districts) plus Jhenaidah Sadar upazila. The primary economic activities are agriculture or small-scale business. The health system consists of a modern 100-bed hospital at the district headquarters, with 31-bed hospitals also existing in each of the five sub-districts. The district and upazila hospitals have both normal delivery and caesarean-section facilities. There is also a district-level Maternal and Child Welfare Centre (MCWC) operating exclusively for providing reproductive-health services, including complicated deliveries. The district is fairly flat terrain, but rain and flooding can make roads impassable throughout the year. As in Uganda, women were selected from all upazila areas to achieve a geographic spread of respondents across the district.

A comparative approach has been used here for presenting findings of interviews in these two settings. Four key areas, which can influence the use of a health facility, were identified, and insights from women in both countries are given into these areas. The four key areas are: (a) initial choice of delivery site; (b) decision-making and decision-makers; (c) overcoming key access barriers; and (d) social norms of delivery.

## RESULTS

### Initial choice of delivery site

One of the most notable differences recognized when comparing the experiences of women in Bangladesh and Uganda was around the initial decision to use health facilities. In Uganda, delivery in health facility among service-users was commonly planned in advance. Twenty-eight of the 30 women interviewed planned to deliver in the facility, while only two women attempted to deliver at home first. In Bangladesh, eight women went to facilities in the first instance, while 22 women attempted to deliver at home first.

As far as the reason for initial decisions, many Ugandan women spoke of pregnancy-related risks and the need to be in a facility in case of complications: 14 women explicitly stated that risk was a reason to use facilities. While the dangers of pregnancy have been identified by both users and non-users in Uganda (18), our interviewees specifically linked these risks to the ability of professionals to manage complications. Some excerpts from the Uganda case-study reports illustrate this view (it is worth noting again that these are not verbatim transcripts but first-person case notes. Facility and names have been removed. Each respondent was anonyomized by assigning a code to the data as follows: IDI standing for in-depth interview; a country code (UG for Uganda, BG for Bangladesh); and a number from 1 to 30 (for record-keeping purposes):

IDI#UG10—20 years old, Catholic, first pregnancy, secondary education: “I also prefer going to Health Centre A because it has a doctor, you never know, in the case of a problem, one can be sure of a doctor's help.”

IDI#UG13—33 years old, Protestant, second pregnancy, partial primary education: “It is… better to deliver in a health centre because a woman can get immediate professional attention, especially if there are complications with the pregnancy.”

These excerpts show how the fear of pregnancy and delivery-related complications was an important driving force for the Ugandan women to decide to use health facilities for delivery. In contrast, the Bangladeshi women's views of pregnancy and birth-related complications did not justify the use of professional care in the first instance. There were only a few exceptions to this, such as in two cases where women had lost children delivering with TBAs in the past (IDI#BG19, BG29), or one case where a woman's sister had lost a child during home-delivery (IDI#BG14). In most Bangladeshi cases, however, the decision to use a facility was only made after women experienced some problems in delivery, such as delivery taking longer than expected or excess bleeding. The women in Bangladesh did not present the view that it was professional medical workers who were best placed to manage delivery-related complications as seen in Uganda. This, however, may be linked to the fact that Bangladeshi women sought care from a large number of alternative healthcare providers when problems were perceived. Treatments were sought from spiritualists, herbalists, TBAs, or local unqualified ‘village doctors’. In such situations, it may be harder to build a consensus around which care-provider is best placed to manage pregnancy and childbirth-related risks.

Interestingly, 22 (73.3%) of the Bangladeshi respondents also reported receiving antenatal care during pregnancy. This compares with a national average of only 37% (21). This could indicate a tendency of these women to engage with services more, which may have influenced the decision to seek professional care, although the initial decision was to attempt delivery at home. Alternatively, it could be a reflection of how antenatal care may serve as a source of information for women to draw upon if problems arise. These possibilities could be investigated in further work.

### Decision-making and decision-makers

Past literature has highlighted the importance of key actors in decisions to use delivery-services, such as the husband in Uganda (11) or mothers and mothers-in-law in Bangladesh (13, 20). The women interviewed in both the countries similarly illustrated the importance of such individuals. In Uganda, some women stated that their husbands made the decision to use a facility. Several women also said that they made the decision themselves, illustrating the decision-making ability of some women. Joint decisions were also, at times, mentioned and, in one case, the woman stated that her mother made the decision (IDI#UG11).

In Bangladesh, the women's explanations of their care-seeking illustrated a much larger number of individuals playing roles in the decision to seek professional care. Decisions were typically made at a crisis point, when a woman's home labour was perceived to be progressing poorly. Close familial actors in the common household, including mothers-in-law, and other female in-laws often gave opinions on how to proceed, yet other actors outside the household could play important roles as well. In one case of a 25-year old woman in her first pregnancy, it was a local teacher's wife who helped convince the family to use a health facility and even lent the family a small amount of money to cover the costs involved (IDI#BG26). In another case, a woman explained how her brother, who was a police officer, convinced her husband to take her to a facility (IDI#BG3). Having personal links to friends or family who worked in a health facility also facilitated the decision to seek care in some cases.

A particularly notable finding from Bangladesh was women's reports of the decision-making roles played by non-medical health practitioners. In many cases, women were attended by TBAs, but ‘village doctors’ (non-qualified individuals who purport to practise western medicine) were also called in when pregnancy was seen to be progressing poorly. In 10 cases, women reported consulting a ‘village doctor’, and, in every case, he supported the decision to take the woman to a health facility. The TBA's roles were more mixed, as some encouraged the use of facilities when they faced problems, while others opposed the decision to go to a facility. The following excerpt illustrates an example of the roles played by multiple individuals both within and outside the family:

Case IDI#BG24—16 years old, Muslim, first pregnancy, primary education: “[The woman's] father contacted the village doctor since delivery was [delayed]. The village doctor examined the respondent and advised to take her to health centre B. But the respondent's mother called in a birth attendant who opposed to take the respondent to the service centre…. When the village doctor was again called in, he expressed his anger for not taking the respondent to the health centre and asked to take the respondent to health centre B immediately. After that, the respondent's mother decided to take the respondent to health centre B as per the advice of the village doctor.”

It was observed that the unqualified ‘village doctors’ often have links to health facilities. In one case, a woman reported that the village doctor arranged deliveries for women in a public facility for some payment (IDI#BG19), while, in another case, a women explained how the village doctors actually owned a private health clinic which employed professional medical staff (IDI#BG18). In cases where these non-qualified practitioners have financial links to professional facilities, they may play an active role in encouraging women to seek professional care—although in cases where women were referred to private facilities, they faced much higher costs compared to those in public facilities.

### Overcoming key access barriers

The two countries also illustrated different ways families could overcome key access barriers, such as costs of care or distance and transportation problems. In the Ugandan cases, it was typically the woman's husband who made arrangements to address these barriers, although parents could also be called upon for needed funds. The Ugandan women often stated how husbands put money aside in advance for clothes or basic medical supplies, illustrating how pre-planning to use facilities could be important to enable families to save up for the event—as childbirth is one of the rare cases where one can predict when health expenditure will occur.

In the few Bangladeshi cases where there was a plan to use facilities in the first instance, the women did not speak of any pre-planning around costs. One woman stated how her husband borrowed money from an uncle and called for transportation to be brought when she was in labour (IDI#BG29). In another case, the woman explained how her family did not expect any costs to be incurred. Only when compelled to purchase supplies for the delivery, her father had to borrow from his brother and then again from a neighbour at a high interest rate (IDI#BG14). For those Bangladeshi women delivering at home in the first instance, a range of different means of raising funds were used for meeting costs. Some wealthier families did not seem to need to take special efforts, but others had to save money in advance, or to sell belongings for money. Family members, such as husband, parents, and in-laws, were most often mentioned as making efforts to raise funds, but, at times, money had to be obtained from outside the household, where it was commonly borrowed at high interest rates (exact rates were not specified). Money could be borrowed from neighbours in this way, or known money lenders, and there were even cases of close relatives also charging interest.

Aside from costs, the Ugandan cases illustrated how transportation difficulties could also be overcome through pre-planning, as the following case-excerpt demonstrates:

IDI#UG14—20 years old, local religion, second pregnancy, some primary education: “My husband would hire a [small] motorcycle to take me for antenatal care or sometimes he would take me on his bicycle. My husband's friend would sometimes tell us to put fuel in the motorcycle, and he would take me to the health centre. At nine months, I bought petrol and kept it in the house. It is the [fuel] we put in my husband's friend's motorcycle to [reach] the health centre.”

The Bangladeshi women primarily reported being transported to health facilities by pedal-driven vehicles. This was in contrast to Uganda where many reported travelling on small motorcycles (locally known as ‘boda-bodas’). Typically, the Bangladeshi women travelled by a ‘van’—a bicycle pulling a flat platform that several people can sit on. Some journeys by this method were of considerable distance—over 10 km, or over an hour in duration—, and several cases explained the difficulties faced with transportation. One case reported travelling two hours over 11 km by a ‘van’ to reach a health facility (IDI#BG13), while another woman had to walk in the rain at night one km over a muddy road to reach a market where a ‘van’ was available (IDI#BG17). The Ugandan women also recognized that transportation might be problematic for women in their community, but only one woman stated how, because she could not get transport in the middle of the night, she went to a nearby health centre, rather than the hospital as planned (IDI#UG27).

A final known barrier to the use of health facilities by women can be difficulties in taking time away from their household responsibilities. In a number of Ugandan cases, husbands reportedly assisted in tasks that their wives would normally undertake, such as housework or cooking. Such support from husbands was not universal, and other friends and family also gave such assistance, but it stood out as particularly enabling women to overcome known barriers in some cases. In contrast, the husband was only one of several possible family members who could play a key role in overcoming access barriers in Bangladesh and was not seen to take over household responsibilities as was the case in Uganda. In most Bangladeshi cases, it appears to have been other relatives—most likely women—who provided assistance to perform household responsibilities when needed.

### Social norms of delivery

The final key area presented here which influences the use of services is that of social norms around facility-use. Despite past studies indicating that facility-use might be stigmatized, none of women interviewed in either country discussed any negative feelings from community members from their decision to use professional services. In Uganda, a number of women reported that their friends also used facilities for delivery. This is despite the fact that a minority of Ugandan women actually deliver in health centres (even when divided by age-group) as the national surveys indicate (9). There is, of course, some potential for respondent bias, where interviewees state this to be the norm simply because they believe it to be the ‘correct’ answer, but another explanation could be a potential ‘clustering’ of users in the district. It may be that, rather than seeing users spread equally across the district, there may be small local communities or social groups for whom facility-use is the norm. Future work could investigate this dynamic to see if, indeed, there are clusters of service-users, identifying possible explanations for such clustering.

In Bangladesh, women did not report any norms of facility-use, and service-users also did not report any social fall-out from their use of facilities either. Part of this may result from the fact that women did attempt a home-delivery at first in most cases. The interviewees opined that facilities were acceptable when complications arose. Past studies have reported experience of delivery-related complications as a key determinant for using professional delivery care (20), and anthropological works have shown how multiple healing practices are acceptable in Bangladesh when a woman's health is at stake—with the best option seen as that which is most effective (22). It would appear that Bangladeshi women can use health facilities, but it may take the idea of a ‘complication’ to justify its use in a social environment valuing independent home-birth.

## DISCUSSION

Both Uganda and Bangladesh, like many low-income countries, have a minority of women giving birth in health facilities and with skilled birth attendants. The decision-making process to do so is a complex one, involving a number of individuals and a need to overcome several barriers—both tangible, such as costs or distance, and social. This comparative study has highlighted some ways women and their families act to overcome such barriers to use professional facilities for childbirth.

Pre-planning was one of the key factors that enabled the Ugandan women to overcome the barriers that existed, with several women commenting on how transportation could be very difficult for other women. Despite remote rural conditions and a hilly terrain, the Ugandan women did not present many stories of problematic transport experiences, unlike in Bangladesh where several women gave quite harrowing tales of difficult trips to facilities decided upon at the last minute. In Bangladesh, it would further appear that individuals are less knowledgeable about what is needed to use facilities, perhaps due to the lower overall use-rates. Some women, however, reported pre-planning for delivery-related expenses, indicating some ability to pre-arrange funds.

Outreach and information about births at facility should include an estimate on expected costs women may face, including a possible acknowledgement of tips and informal payments often charged for supplies (although the national policy is for public services to be free, it is clear that women face some expenses which are often known to families). Encouraging families to plan for cost and availability of transport is also important, and such information could potentially be provided when pregnant women receive tetanus toxoid immunization, as over 80% of Bangladeshi women receive at least one dose during pregnancy (21). Currently, in Bangladesh, only a few small-scale interventions are ongoing to promote birth-planning, which could, in theory, be scaled up if a need was felt nationally (23, 24). Any improved education messages in Bangladesh will also need to engage with multiple approaches to healing women can take. Building a consensus that professional health facilities are best suited to address the risks of childbirth needs to be a central message, as this appears to underlie an initial decision many Ugandan women to deliver in a facility. Finally, increasing antenatal care-use among Bangladeshi women may also be important to influence later decisions for birth at facility. While international literature has turned its focus away from antenatal risk screening due to the inability to predict complicated cases (25, 26), antenatal care may provide women with some knowledge of health facilities so that when problems arise at home during labour, they may be more willing or able to seek professional care.

In terms of key decision-makers and overcoming access barriers, experiences in both the countries have pointed to the importance of social networks. Women do not seek care individually for pregnancy and childbirth, but rely on a supportive social environment both for decision-making and for actions to use care. In the Ugandan cases, it was almost always the husband or close family networks who supported the decision and action to use a facility for delivery. However, several women's reports that their friends and community members also deliver in facilities points to a potential clustering of users, emphasizing the role of community social networks in setting norms of facility-use. This finding would indicate that policy-makers may look to engage with community-based women's groups and networks promote skilled attendance, rather than focussing solely on individually-oriented education campaigns.

Our Bangladeshi cases illustrate how, at crisis points, members of women's ‘extended’ social networks (beyond close friends or family members) may play greater roles than other situations, such as early birth-planning, where the core members of the family normally dominate decision-making (27). Having broader social network links to key individuals with knowledge of, or links to, facilities may be particularly useful to facilitate decision-making, including links through alternative health providers.

Overall, this study points to the complexity of factors enabling or preventing the use of professional delivery services, highlighting the need for policy-makers to engage with what have been termed ‘health-enabling communities’ (28), including the wider network of individuals influential in decision-making and providing support to overcome access barriers. Early planning around the use of services can be particularly useful to help overcome technical access barriers, such as costs or transportation, but it is also essential to engage with individuals through their social networks to influence both decision-making and resources for seeking care. Only by addressing these issues can policy-makers effectively work to increase the number of deliveries in facilities in resource-constrained settings.

## ACKNOWLEDGEMENTS

The authors are members of the Health Systems Development Programme which is funded by the Department for International Development (DFID), UK. The DFID supports policies, programmes, and projects to promote international development. It provided funds for this study as part of that objective, but the views and opinions expressed are those of the authors alone.
